# Efficiency of Recombinant CRISPR/rCas9-Mediated miRNA Gene Editing in Rice

**DOI:** 10.3390/ijms21249606

**Published:** 2020-12-16

**Authors:** Pil Joong Chung, Hoyong Chung, Nuri Oh, Joohee Choi, Seung Woon Bang, Se Eun Jung, Harin Jung, Jae Sung Shim, Ju-Kon Kim

**Affiliations:** 1 Graduate School of International Agricultural Technology and Crop Biotechnology Institute/GreenBio Science & Technology, Seoul National University, Pyeongchang 25354, Korea; chungpj@gmail.com (P.J.C.); hychung@3bigs.com (H.C.); 0901nuri@gmail.com (N.O.); joohit03@gmail.com (J.C.); seungwoon93@gmail.com (S.W.B.); seun0204@snu.ac.kr (S.E.J.); harin0723@gmail.com (H.J.); 2 Temasek Life Science Laboratory, National University of Singapore, Singapore 117604, Singapore; 3 3BIGS, Suwon 16506, Korea; 4 College of Agriculture and Life Sciences, Seoul National University, Seoul 08826, Korea; 5 Novel food Division, National Institute of Food and Drug Safety Evaluation, Cheongju 28159, Korea; 6 NUS Synthetic Biology for Clinical and Technological Innovation, Department of Biochemistry, Yong Loo Lin, School of Medicine, National University of Singapore, Singapore 117596, Singapore; 7 School of Biological Sciences and Technology, Chonnam National University, Gwangju 61186, Korea

**Keywords:** CRISPR/rCas9, drought-responsive miRNAs, INDELs, *Oryza sativa*, rice protoplast, sgRNAs

## Abstract

Drought is one of the major environmental stresses adversely affecting crop productivity worldwide. Precise characterization of genes involved in drought response is necessary to develop new crop varieties with enhanced drought tolerance. Previously, we identified 66 drought-induced miRNAs in rice plants. For the further functional investigation of the miRNAs, we applied recombinant codon-optimized Cas9 (rCas9) for rice with single-guide RNAs specifically targeting mature miRNA sequences or sites required for the biogenesis of mature miRNA. A total of 458 T_0_ transgenic plants were analyzed to determine the frequency and type of mutations induced by CRISPR/rCas9 on 13 independent target miRNAs. The average mutation frequency for 13 genes targeted by single guide RNAs (sgRNAs) in T_0_ generation was 59.4%, including mono-allelic (8.54%), bi-allelic (11.1%), and hetero-allelic combination (39.7%) mutations. The mutation frequency showed a positive correlation with Tm temperature of sgRNAs. For base insertion, one base insertion (99%) was predominantly detected in transgenic plants. Similarly, one base deletion accounted for the highest percentage, but there was also a significant percentage of cases in which more than one base was deleted. The deletion of more than two bases in OsmiR171f and OsmiR818b significantly reduced the level of corresponding mature miRNAs. Further functional analysis using CRISPR/Cas9-mediated mutagenesis confirmed that OsmiR818b is involved in drought response in rice plants. Overall, this study suggests that the CRISPR/rCas9 system is a powerful tool for loss-of-function analysis of miRNA in rice.

## 1. Introduction

Targeted genome editing with site-directed nucleases (SDNs) is a promising tool for both basic and applied biological research. The SDNs, including zinc-finger nucleases (ZFNs), transcription activator-like effector nucleases (TALENs), and the clustered regularly-interspaced short palindromic repeats (CRISPR)-associated 9 (Cas9) system, induce targeted DNA double-strand breaks (DSBs) and subsequently trigger DNA repair through non-homologous end-joining (NHEJ) or homologous recombination (HR) pathway [1,2,3]. SDN-mediated genome editing facilitates the development of transgene-free new crop varieties because the changes of the genome sequence are able to be separated from the transgene.

Among three representative SDNs, CRISPR/Cas9 is regarded as an efficient system to introduce mutations and gene fragments into the plant genome [3,4]. The CRISPR/Cas9 system is composed of a Cas9 endonuclease, a CRISPR RNA (crRNA), and a trans-acting crRNA (tracrRNA) isolated from *Streptococcus pyogenes* [1]. A crRNA is responsible for targeting a specific DNA site, and a tracrRNA provides a scaffold that is recognized by a Cas9 protein. The crRNA is base-paired to tracrRNA and form a two-RNA structure that directs the Cas9 to the target site [5]. A chimeric single-guide RNA (sgRNA), which is a fusion of a CRISPR RNA (crRNA) and a trans-acting crRNA (tracrRNA), also successfully directs Cas9 for sequence-specific DNA cleavage [6]. The CRISPR/Cas9 system recognizes 20 specific nucleotides followed by a protospacer-adjacent motif (PAM) sequence and cleaves DNA at approximately three base pairs (bps) upstream of the PAM sequence. During the repair processes, variable sizes of base insertions and deletions (indels) occur at target sites, resulting in site-specific changes of genetic information. Thus, the proper design of sgRNA is sufficient to induce mutations on the specific location of the genome. In addition, the CRISPR/Cas9 system can simultaneously edit multiple target sites by using multiple gRNAs encoded in a single CRISPR array [7]. Based on these advantages, the CRISPR/Cas9 system is extensively used for the functional characterization of genes, especially in plants [4,7,8].

microRNA (miRNA) is a class of small non-coding RNAs that directly regulate the functions of specific messenger RNAs through transcriptional or translational repression [9,10,11]. miRNA is transcribed into a form of primary miRNA and processed into 20–24 nt small mature miRNAs through the actions of multiple components such as Dicer-like RNase III endonucleases (DCLs) [12]. Gain- and loss-of-functional approaches are the most effective strategies to characterize the function of a particular gene [13]. Given the nature of miRNA that is transcribed from the genome, precise determination of a transcript unit is required for gain-of-functional approaches by overexpressing it. Artificial RNA can be also directly expressed under the control of appropriate promoters [13,14]. On the other hand, loss-of-function analysis of miRNA is difficult due to the lack of appropriate knockout mutants. Loss-of-function mutation of miRNA is difficult to achieve by insertional mutagenesis due to the small sequence size and insufficient coverage of inserts in rice. For this reason, post-transcriptional approaches that sequester or degrade miRNAs have been used for the loss-of-function analysis of miRNA [15,16,17], but these approaches often produced considerable variations in the degree of inhibition on miRNA function [18]. Recently, CRISPR/Cas9 has been spotlighted as an alternative option to overcome these limitations [4,19]. Here, we aimed to generate a loss-of-function mutant of 12 independent miRNAs related to drought responses in rice using miRNA-specific sgRNA [20] and recombinant codon-optimized Cas9 (rCas9) for rice. The large-scale analysis of transgenic plants expressing the CRISPR/rCas9 system provides technical and biological information that is helpful for reversing genetic approaches for the functional analysis of miRNA using the CRISPR/rCas9 system in rice.

## 2. Results

### 2.1. Functional Validation of Rice Codon-Optimized rCas9 Using Rice Protoplasts

**Figure 1:**
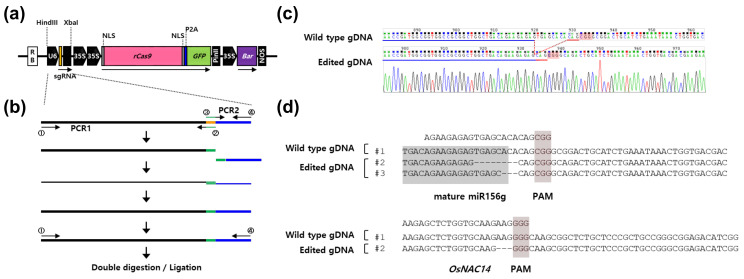
Construction of binary vectors and confirmation of clustered regularly-interspaced short palindromic repeats (CRISPR)/ recombinant codon-optimized Cas9 (rCas9) system in rice protoplasts. (**a**) Diagram of recombinant CRISPR/rCas9 constructs. A rice codon-optimized *Streptococcus pyogenes* Cas9 *(rCas9)* fusing with N-terminal and C-terminal nuclear localization signal (NLS), self-cleaving 2A peptide (P2A), and GFP were inserted downstream of dual *Cauliflower mosaic virus 35S* (*CaMV 35S*) promoters. The rice *U6* promoter and single-guide RNA (sgRNA) were cloned between the right border (RB) and *CaMV 35S* promoters. Bar, phosphinothricin *N*-acetyltransferase; PinII, potato proteinase inhibitor II terminator; NOS, nopaline synthase terminator; LB, left border driven by 35S promoter. (**b**) Construction of the *pU6:sgRNA* cassette using overlapping PCR. To introduce a specific sequence between the *U6* promoter and guide RNA scaffold sequence, a specific sequence was incorporated at the 5′ ends of guide RNA scaffold specific forward primer (③). The reverse complementary sequence of the specific sequence was added at the 5′ end of the *U6* promoter-specific reverse primer (②). Two PCR products (PCR1 and PCR2) were annealed together through base pairing between the inserted specific sequences, and each strand was extended by a sequence that was complementary to the sequence it was to be joined to, producing a dsDNA template. An additional round of PCR was carried out to amplify the pU6:sgRNA cassette. The final PCR product was inserted into a CRISPR/rCas9 vector through *Hind*III and *Xba*I restriction sites. (**c**) The rice protoplasts were transfected with a CRISPR/rCas9 vector containing sgRNA specific for *OsmiR156g*. A representative chromatogram was obtained from direct PCR sequencing analysis of the transfected protoplasts. The mutated target region is indicated with red dotted line. The protospacer adjacent motif (PAM) site is underlined in red. (**d**) The rice protoplasts were transfected with a CRISPR/rCas9 vector containing sgRNA specific for OsmiR156g or OsNAC14. Genomic DNA extracted from the protoplasts was used for amplification of the genomic region targeted by sgRNA by PCR reaction. The PCR products were then subcloned into the TA cloning vector for sequencing analysis. The mature miR156g sequence and PAM site are highlighted with gray and red boxes, respectively.

To accommodate the CRISPR/rCas9 system in rice, we constructed a binary vector expressing single guide RNA (sgRNA) under the control of rice *U6* promoter and recombinant codon-optimized *Streptococcus pyogenes Cas9* (*rCas9*) under the control of *CaMV 35S* promoter (pSB11_U6::sgRNA-35S::rCas9) (Figure 1a). The target-specific gRNA sequence was inserted between the rice *U6* promoter and the sgRNA scaffold by overlapping PCR (Figure 1b). To test whether the recombinant CRISPR/rCas9 system worked properly and could be used to specifically modify an endogenous genome in rice, we designed two sgRNAs specifically targeting the *OsmiR156g* and *OsNAC14* locus, respectively. The final constructs were transiently expressed in rice leaf protoplasts. To identify mutations induced by transient expression of the CRISPR/Cas9 system, sgRNAs targeting genomic regions were analyzed by cloning PCR products into the TA cloning vector followed by sequencing (Figure 1c). Sequencing analysis revealed that de novo mutations were generated on the *OsmiR156* gene targeted by sgRNA, and two different deletion mutations were identified in which three or eight bases were removed out of 10 sequencing results (Figure 1d). Similarly, three base pair deletions were found from the *OsNAC14* locus targeted by sgRNA (Figure 1d). The deletions were located in close proximity to the predicted cleavage site of the Cas9/sgRNA complex (three to four bps downstream of the PAM sequence or immediately adjacent to the PAM sequence), indicating that the deletions of target sequences could be caused by rCas9-directed DNA cleavage and followed the non-homologous end-joining repair pathway.

### 2.2. Genome Editing Patterns Generated by rCas9

**Figure 2:**
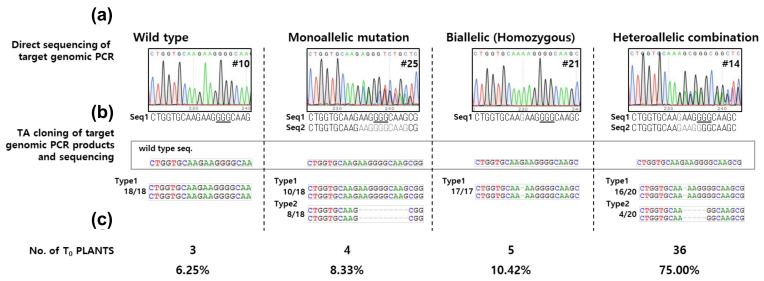
Confirmation of the CRISPR/rCas9 system in rice. Genomic DNA was extracted from the 48 T_0_ transgenic plants expressing *rCas9* and *sgRNA* specific for *OsNAC14* and used for sequencing analysis. (**a**) Representative chromatograms obtained from the sequencing analysis of PCR products. (**b**) Type of mutations on the sgRNA target site determined through TA-cloning-coupled sequencing analysis. (**c**) The number of T_0_ plants showing an indicated type of mutation. Wild type, non-mutated transgenic plants; monoallelic mutation, only one allele is mutated; biallelic, the two alleles are mutated; homozygous, the two alleles have the same mutations; heterozygous, the two alleles have different mutations.

To confirm that stable expression of rCas9 also induces mutation in rice, we generated transgenic rice plants expressing rCas9 under the control of *35S* promoter and *OsNAC14*-specific sgRNA under the control of *U6* promoter through Agrobacterium-mediated transformation (*CRISPR/rCas9^OsNAC14^*). Mutation patterns in the sgRNA targeting site were confirmed by targeted sequencing analysis of PCR products (Figure 2a and Figure S1). The accuracy of the targeted sequencing method was further confirmed by TA-cloning-coupled sequencing of the sgRNA targeting region (Figure 2b). The sequencing analysis of the 48 T_0_ transgenic plants revealed that the CRISPR/rCas9 system successfully induced mutations on the sgRNA targeting site. Among the analyzed 48 T_0_ plants, 45 transgenic plants contained indels at the target sites, four transgenic plants contained monoallelic mutations, and 41 transgenic plants contained biallelic or heteroallelic combination mutations. Various types of mutations were found in the analyzed *CRISPR/rCas9^OsNAC14^* plants. Two bases were frequently deleted in the analyzed *CRISPR/rCas9^OsNAC14^* plants (Figure S2). In the case of base insertion, one base insertion was predominantly detected from the 48 plants. These results indicate that CRISPR/rCas9 can be used for site-specific mutagenesis in rice.

### 2.3. The Efficiency of CRISPR/rCas9-Mediated Mutagenesis on miRNA Genes

To precisely evaluate the efficiency of rCas9-driven mutation, we chose 12 rice drought-responsive miRNAs as targets for CRISPR/rCas9-mediated mutagenesis. A total of 458 T_0_ transgenic rice plants was used for analyzing mutation frequency and indel patterns on the sgRNA-targeting sites. Sequencing analysis showed that 59.4% (272 out of 458) of transgenic plants contained mutations on their target sites (Table 1). The highest mutation efficiency was 100% for *miR399K* and the lowest mutation efficiency was 3.0% for *miR816*. The majority of mutation types were biallelic mutations (50.8%), and 8.5% of the transgenic plants contained monoallelic mutations. Among biallelic mutations, homozygous and heterogeneous mutations accounted for 21.9% and 78.1%, respectively.

Mutation data were obtained from sequencing analysis of a total of 458 T_0_ transgenic plants transformed with CRISPR/rCas9. sgRNA, target sequence (black) and PAM sequence (red); GC (%), GC content of 20 bp sgRNA sequence; Tm, melting temperature of 20 bp sgRNA sequence; WT, non-mutated transgenic plants; mono-allelic, only one allele is mutated; bi-allelic, the two alleles are mutated; homo, the two alleles have the same mutations; hetero, the two alleles have different mutations; total plants, number of transgenic plants used for the analysis; mutation rate (%), the ratio of transgenic plants with mutations from total transgenic plants.

### 2.4. Patterns and Position of CRISPR/rCas9-Mediated Mutations in T_0_ Transgenic Rice

**Figure 3:**
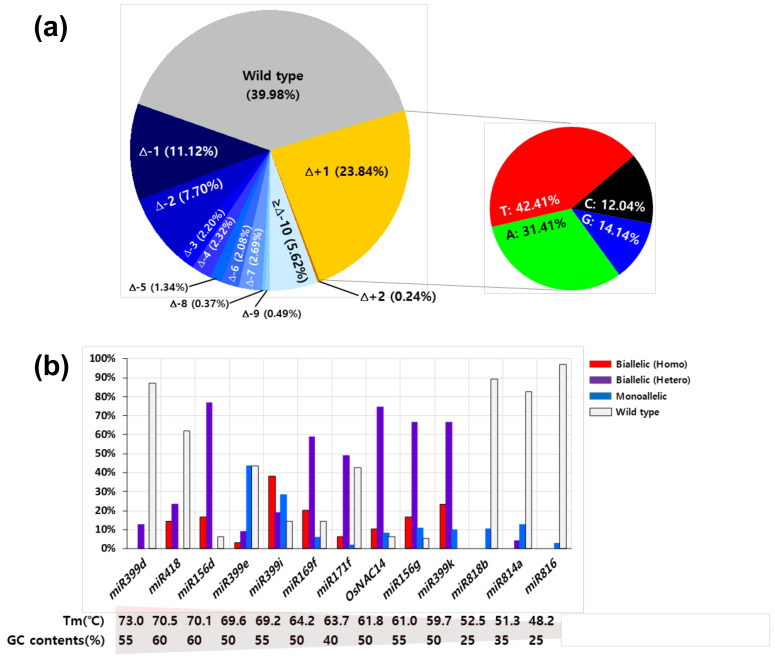
Mutation efficiency and type in T_0_ transgenic rice plants expressing CRISPR/Cas9. Sequence information of the sgRNA targeting site was collected from 458 T_0_ transgenic rice plants. (**a**) The pie chart displays the frequency of each mutation type found in 458 T_0_ transgenic rice plants. (**b**) The bar chart indicates the proportion of mutation types in transgenic plants. Tm, melting temperature of 20 bp sgRNA sequence; GC content, GC content of 20 bp sgRNA sequence; bi-allelic (homo), the two alleles have the same mutated (red bar); bi-allelic (hetero), the two alleles have different mutations (purple bar); mono-allelic, only one allele is mutated (blue bar); wild type, plants that had not been mutated (White bar).

**Figure 4:**
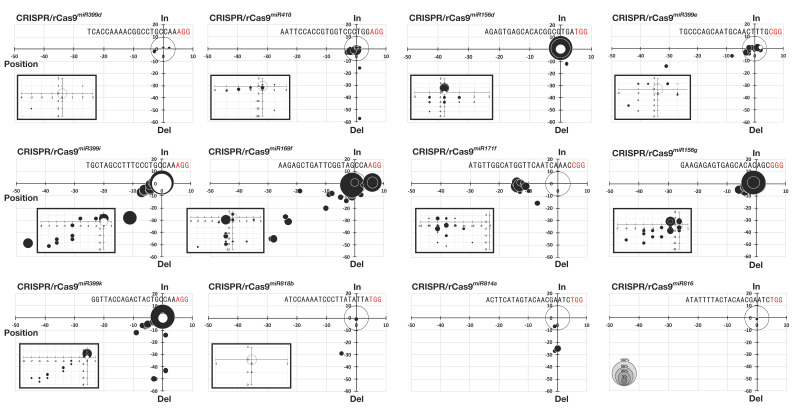
Patterns and positions of CRISPR/rCas9-mediated base insertion and deletion (Iindel) mutations. The graph shows the correlation between the degree of indel (y-axis) and the position where indel occurs (x-axis). The square in the graph is an enlarged image of the area where spots are concentrated. The white circle at the origin indicates the frequency of non-mutated plants.

We then analyzed mutation patterns in the transgenic plants. The base deletions and insertions accounted for 35.9% and 24.1%, respectively (Figure 3a). The proportion of one and two base deletions was 52.4% of total base deletions, and more than three base deletions accounted for 47.6%. In the case of base insertion, 99% of target sites with insertion contained single base insertion (Figure 3a). All four bases were inserted into the plant genome, of which thymine (T) or adenine (A) had a high proportion (73.82%) (Figure 3a). We found a potential correlation between T_m_ temperature and mutation frequency (Figure 3b). Relatively-higher mutation frequency was observed when using sgRNAs with a high Tm temperature than those with low T_m_ temperature except for the *miR399d* locus. In addition, frequent biallelic mutations were observed in plants transformed with sgRNAs with high Tm temperatures (Figure 3b). On the other hand, monoallelic mutations were predominantly observed in transgenic plants expressing sgRNAs with Tm temperatures lower than 40% (Figure 3b). Next, we analyzed the correlation between the size of the indel and the position of the mutation (Figure 4). Differently from mutations observed in protoplasts, mutations occurred on not only three bases upstream of the PAM site but also other positions in the transgenic plants. Moreover, several plants carried mutations on the PAM site or even downstream of the PAM site (Figure 4). Large deletions of more than 10 bases were observed in CRISPR/rCas9*^miR41^*^8^, CRISPR/rCas9*^miR156d^*, CRISPR/rCas9*^miR399e^*, CRISPR/rCas9*^miR399i^*, CRISPR/rCas9*^miR169f^*, CRISPR/rCas9*^miR171f^*, CRISPR/rCas9*^miR399k^*, and CRISPR/rCas9*^miR814a^* transgenic plants. The longest deletion detected was 68 base deletions in CRISPR/rCas9*^miR418^* transgenic plants (Figure 4). These results indicate that the CRISPR/rCas9 system could induce various types of deletions that would be beneficial for the effective elimination of miRNA functions.

### 2.5. Inheritance of rCas9-Mediated Mutation

**Figure 5:**
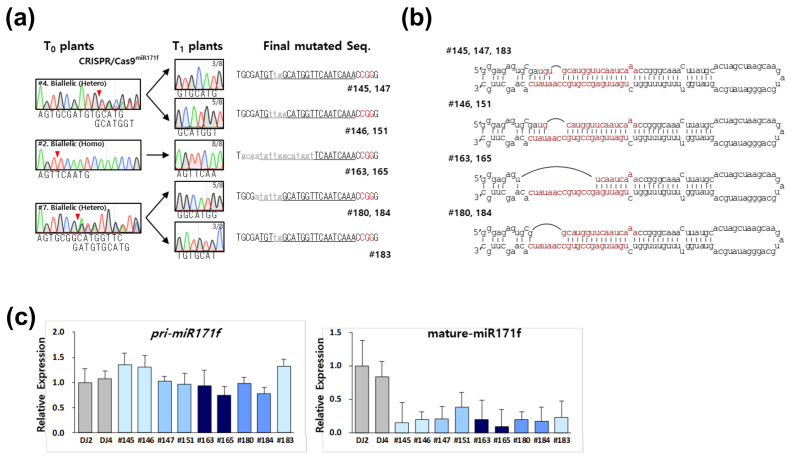
Characterization of CRISPR/Cas9*^miR171f^* T_1_ plants with homozygous mutations. (**a**) Mutation patterns were determined from CRISPR/Cas9*^miR171f^* T_1_ plants. T_1_ generation obtained from three independent CRISPR/Cas9*^miR171f^* transgenic plants was used to investigate the inheritance and segregation of mutations observed in the T_0_ stage. (**b**) Prediction of the stem–loop structure of the mutated *miR171f*. The mature miR171f is shown in red. (**c**) Expression patterns of *pri-miR171f* and mature miR171f-5p in non-transgenic and CRISPR/Cas9*^miR171f^* transgenic plants.

To confirm the stable transmission of rCas9-mediated mutation in T_0_ plants into its T_1_ siblings, we chose transgenic plants carrying three different forms of mutations on *miR171f*. CRISPR/rCas9*^miR171f^* #4 transgenic plants contained biallelic mutations composed of deletion of two or four bases in the T_0_ stage. In the T_1_ stage, three plants were homozygous mutants with two base deletions while five plants turned out to be homozygous mutants with four base deletions **(**Figure 5a). Similarly, biallelic mutations in the T_0_ stage of *CRISPR/rCas9^miR171f^* #7 transgenic plants were segregated in the T_1_ stage. In addition, *CRISPR/rCas9^miR171f^* #2 transgenic plants carrying a homozygous mutation in the T_0_ stage also had a homozygous mutation in the T_1_ stage (Figure 5a). These results indicate that mutations observed in transgenic plants in the T_0_ stage are successively inherited into the next siblings.

Differently from a gene that codes protein, miRNA functions as an RNA, thus insertion or deletion of bases is insufficient to guarantee functional impairment of a miRNA. To evaluate the effect of rCas9-driven mutations on the function of miRNAs, we first illustrated the position of mutations on the *pre*-*miR171f* sequence (Figure 5b). All four mutations were located on the mature *miR171f* sequence. The mutations did not affect transcription of *pre*-*miR171f* transcripts as evidenced by qPCR analysis (Figure 5c). However, the mutations significantly reduced the level of mature *OsmiR171f-5p* (Figure 5c). These results suggest that deletions of mature *miR171* sequence affect its transcript level in rice.

### 2.6. Function of the Drought-Induced OsmiR818b on Drought Responses in Rice Plants

**Figure 6:**
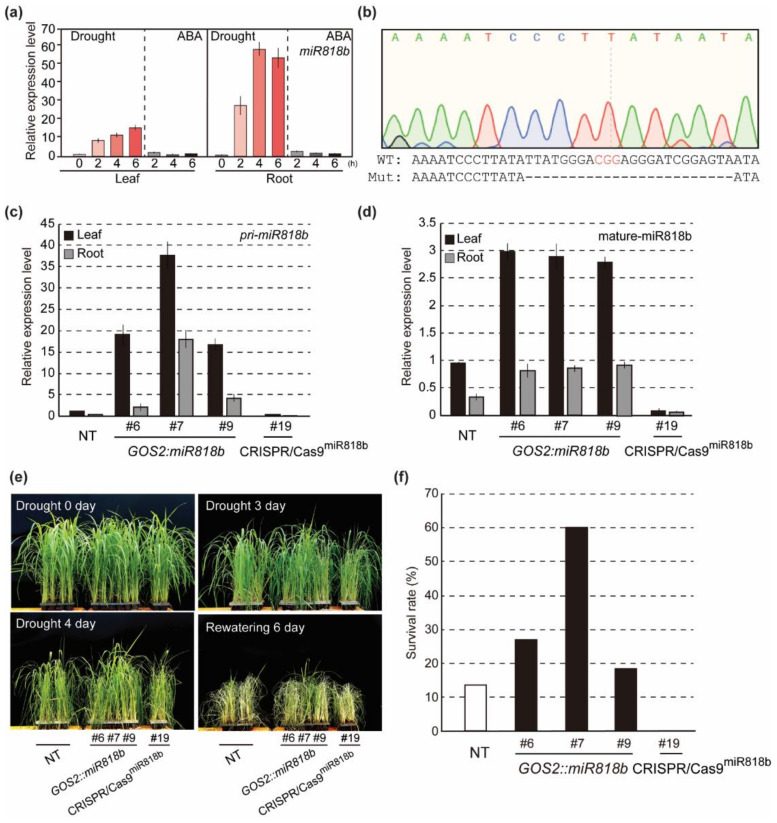
Drought response of *OsmiR818b*-overexpressing and CRISPR/Cas9^miR818b^ plants. (**a**) Expression patterns of *pri-miR818b* in response to drought and abscisic acid (ABA) treatments. Two-week-old non-transgenic rice (*O. sativa* cv. Dongjin) seedlings were treated with drought and 100 μM ABA. The plants were harvested at the indicated time points after treatments. (**b**) Mutation pattern on *OsmiR818b* sequence in CRISPR/Cas9^miR818b^ #19 transgenic plants. (**c**) Expression level of *pri-OsmiR818b* and (**d**) mature-OsmiR818b in *OsmiR818b*-overexpressing (*GOS2:miR818b*) and CRISPR/Cas9^miR818b^ #19 transgenic plants. (**e**) The responses of the transgenic plants during drought treatments. One-month-old non-transgenic (NT), *OsmiR818b*-overexpressing (*GOS2:miR818b*) and CRISPR/Cas9^miR818b^ #19 mutant plants were exposed to drought stress for 4 days, followed by re-watering for 6 days. (**f**) The survival rate of the transgenic plants 6 days after re-watering (*n* = 30).

To confirm that the CRISPR/Cas9-mediated mutagenesis can be used for functional analysis of miRNAs, we used the drought-responsive OsmiR818b for further analysis. We first investigated expression patterns of *OsmiR818b* in response to drought. *OsmiR818b* expression was induced by drought treatments in both leaves and roots of rice plants (Figure 6a). On the other hand, *OsmiR818b* expression was rarely changed by abscisic acid (ABA) treatments (Figure 6a). These data indicate that *OsmiR818b* expression is induced by drought in an ABA-independent pathway. To address function of OsmiR818b, we generated *OsmiR818b*-overexpressing transgenic plants (*GOS2::miR818b*) and CRIPSR/Cas9^miR818b^ mutant plants. The CRIPSR/Cas9^miR818b^ #19 mutant plants contained a biallelic mutation composed of a 24 base deletion (Figure 6b). Expression analysis revealed that both primary (pri-) and mature-OsmiR818b were highly expressed in *OsmiR818b*-overexpressing plants while levels of pri- and mature-OsmiR818b was reduced in CRIPSR/Cas9^miR818b^ mutant plants (Figure 6c,d). To investigate effect of the altered expression of *OsmiR818b* on drought response in rice plants, we treated drought stress on *OsmiR818b*-overexpressing and CRIPSR/Cas9^miR818b^ mutant plants as well as non-transgenic (NT) control plants, and monitored drought-induced visual symptoms. Drought induced symptom such as leaf rolling and wilting appeared earlier in CRIPSR/Cas9^miR818b^ mutant plants compared to those in NT control plants (Figure 6e). in addition, CRIPSR/Cas9^miR818b^ mutant plants showed lower recovery rate compared to NT plants after being relieved from drought stress through re-watering (Figure 6f). On the other hand, *OsmiR818b*-overexpressing plants were relatively more tolerant to drought treatment than NT plants (Figure 6e,f). These result suggest that *OsmiR818b* participates in drought response of rice plants and increase of *OsmiR818b* expression improves drought tolerance in rice plants. 

## 3. Discussion

Genome editing efficiency in plants is largely determined by several crucial factors including the expression level of Cas9 and sgRNA [21,22]. Codon optimization is one of the feasible options to increase the translation of recombinant protein in plants. For this reason, several plant codon-optimized Cas9 have been introduced into various plants [23,24]. Here, we tested mutation efficiency using recombinant rice codon-optimized Cas9 (rCas9). Our data suggest that the CRISPR/rCas9 system can induce mutations on target sites with high efficiency in rice (Table 1). rCas9 successfully induced mutations on plant genomes targeted by sgRNA in both transient and stable expression systems (Figure 1 and Figure 2). Sequence analysis of 458 transgenic plants at the T_0_ stage revealed that 59.4% of the tested plants carried monoallelic or biallelic mutations (Table 1). Biallelic mutations were found from 66.9% of the transgenic plants with mutations. In addition, mutation efficiency was greater than 85.0% in 6 out of the 13 tested miRNAs (Table 1). Moreover, the probability of occurrence of homozygous mutations was 11.1% (Table 1). It has been reported that the introduction of *SpCas9* with sgRNA showed a mutation induction rate of 44.4% in rice, and 3.8% of transgenic plants carrying homozygous mutations [25]. Thus, the CRISPR/rCas9 system used in this study can be a tool for efficient targeted mutagenesis in rice.

The mutation frequency was related to the Tm temperature of the target sequences. Target sequences with Tm temperature over 53 °C except for *miR399d* showed significantly-higher mutation frequency than those with low Tm temperature (Figure 3b). A similar correlation between mutation frequency and GC ratio has been reported in several organisms [8,26,27]. It has also been reported that the existence of purine residues at the end of the sgRNA is important for genome editing efficiency [8]. Our analysis showed that the contribution of purine residues in sgRNA on genome editing efficiency seems relatively minor compared to that of Tm temperature.

CRISPR/rCas9-mediated mutations are generally detected three bases upstream of the PAM site because Cas9 binds to sgRNA and cleaves three bases upstream of the PAM site [8]. Consistently, our data showed that all detected mutations generated by transient expression of CRISPR/rCas9 in protoplasts were located three bases upstream of the PAM sequence (Figure 1c,d). However, stable expression of CRISPR/rCas9, even with the same constructs, caused mutations in a wider range of regions including three bases upstream of the PAM site (Figure 2 and Figure 4). It is not clear how these differences were generated between two different expression systems. One possible explanation is that NHEJ activity or stability of truncated double-strand DNA might be different between protoplasts and calluses. Similar to our data, Shan et al. reported that the TALEN system produced longer deletions by stable expression in calluses compared with transient expression in protoplasts of rice and Brachypodium [28]. Further investigations will be helpful to understand the difference in mutation types that occurred by transient and stable expression of molecular scissors.

Strategies for miRNA knockout are essential for studying miRNA function in plants [29]. The major loss-of-function technologies in miRNA research include miRNA-specific antisense inhibitors, miRNA sponges, and genetic knockouts [30]. Among them, a genetic knockout is the most reliable technique to determine the functions of miRNA. Hitherto, the CRISPR/Cas9 system has been exclusively applied in human, mouse, or zebrafish cell lines to knockout miRNA genes [31,32,33,34]. Similarly, various attempts have been reported to create miRNA knockout plants using the CRISPR/Cas9 system for the functional characterization of miRNA in plants [19,29]. Mutations on mature miRNA sequences or flanking regions which are important for miRNA processing are crucial for successful knockout of miRNA. In addition, the deletion of multiple bases will increase the chance of miRNA knockout production. Our data indicate that stable expression of the CRISPR/rCas9 system in rice generated large deletions on miRNA sequences targeted by sgRNAs (Figure 4). For example, the CRISPR/rCas9 system induced 2 to 16 base deletions on the *miR171f* gene (Figure 5a). The deletions changed the level of mature miRNA171f but not the transcription level of *pri-miR171f* (Figure 5c). Moreover, deletion of 24 bases on the miR818b significantly reduced levels of both *pri*- and mature-miR818b (Figure 6c,d). The functional analysis using CRISPR/Cas9^mir818b^ mutant and *miR818b*-overexpressing plants showed that *OsmiR818b* is involved in drought tolerance in rice plants (Figure 6e,f). These data indicate that a deletion within the pre-miRNA regions by CRISPR/rCas9 can be used for functional analysis of miRNAs in rice.

Data presented in this study suggest that early isolation of T_0_ plants with biallelic homozygous and heteroallelic combinational mutations is a useful approach to obtain homogenous mutants in the next generation since the mutation patterns are stably inherited following the classic Mendelian law (Figure 5a). The generation of transgenic plants is one of the major limiting steps for crop research. In this study, we obtained 233 T_0_ lines with the biallelic mutation from 458 T_0_ transgenic plants **(**Table 1), indicating that at least 10 T_0_ plants should be generated to obtain five transgenic plants with biallelic mutations. The efficiency will increase up to 60.6% by using sgRNA with high GC ratio (Table 1). These results indicate that the use of rCas9 and sgRNA with a high GC ratio will greatly accelerate the generation of biallelic mutations on the target sequence in the T_0_ stage which is crucial for the efficient development of genome-edited rice. Further optimization of CRISPR/rCas9 in rice would promise higher mutation efficiency as well as the rapid creation of new varieties with valuable and novel traits.

## 4. Materials and Methods

### 4.1. Plasmid Construction

To apply the CRISPR/rCas9 system in rice, a recombinant codon-optimized *Streptococcus pyogenes* Cas9 for rice and single-guide RNA (sgRNA) were constructed in the pSB11 vector [35] through restriction-enzyme-mediated excision and ligation reactions. The rice codon-optimized *Streptococcus pyogenes* Cas9 was chemically synthesized by Bioneer in Korea (Daejeon, Korea). The expression of sgRNA and Cas9 was driven by the rice U6 promoter and *Cauliflower mosaic virus* 35S (*CaMV* 35S) promoter, respectively. Nuclear localization sequence (NLS) was fused to both N-terminus and C-terminus of the rice codon-optimized rCas9, and self-cleaving 2A peptide (P2A) and GFP were inserted into the C-terminal NLS sequence (Accession number MW296054) (Figure 1a). The 20 bp upstream sequence from the PAM (protospacer adjacent motif) sequence nearest to the miRNA seed sequence was used to guide RNA design.

For replacing gRNA in these vectors, the *U6* promoter (*pOsU6*) and a sequence-specific gRNA were introduced into the pSB11 vector through *Hind*III and *Xba*I sites ((New England Biolabs, Ipswich, MA, USA)) (Figure 1a). To construct the *pOsU6:sgRNA* cassette containing a target-specific guide sequence, the guide RNA sequence was added at the 3′ and 5′ ends of the U6 promoter through three-step PCR reaction (Figure 1b). The final construct was sequenced to verify the correct insertion of the *pOsU6:sgRNA-2×35S:rCas9* cassette. To generate *OsmiR818b*-overexpressing plants, the primary *OsmiR818b* sequence was amplified from rice (*Oryza sativa L.ssp. japonica* cv. Dongjin) total RNAs using the reverse transcription system (Promega, Madison, WI, USA) and PrimeSTAR HS DNA polymerase (Takara, Kyoto, Japan) with gene-specific primers (forward primer: CACCGATCGATCTCGTCGTCG, reverse primer: GAACCTTGCACATGACTTCAGCTAG). The amplified OsmiR818b sequence was cloned into the rice p700 transformation vector harboring GOS2 promoter for constitutive overexpression [36]. The final construct was named as *GOS2::OsmiR818b*. The primer information used for plasmid construction can be found in Table S1.

### 4.2. Transient Expression of CRISPR/rCas9 Using Protoplasts

The preparation of rice protoplasts was carried out as previously described [37]. For transient expression of sgRNA and CRISPR/rCas9, 50 μL of protoplasts (2–3 × 10^6^ cells) was mixed with 15 μL of the CRISPR/rCas9 vector (1–2 μg) and 130 μL of 40% PEG solution (Sigma, St. Louis, MO, USA), and incubated for 15 min at 28 °C in the dark. After incubation, 1mL of W5 solution (154 mM NaCl, 125 mM CaCl_2_, 5 mM KCl, 2 mM MES (pH = 5.7)) was added into the mixture, and the protoplasts were incubated for 12 h at 28 °C. After incubation, the protoplasts were collected and used for genomic DNA extraction. The sgRNA target site was PCR-amplified and cloned into the pGEM-T Easy vector (Promega, USA). Colonies in the LB plates were picked and their insert sequences were determined by the Sanger method using target-specific primers (Cosmogenetech, Seoul, Korea).

### 4.3. Plant Transformation

Transgenic rice plants were obtained by *A. tumefaciens* strain LBA4404-mediated transformation of rice (*Oryza sativa L*. cv. Dongjin) embryonic callus as previously described [38]. The CRISPR/rCas9 or *GOS2::OsmiR818b* constructs were introduced into *Agrobacterium tumefaciens* strain LBA4404 by the triparental mating method with the helper cell.

### 4.4. Analysis of Mutation Frequency in Transgenic Plants by Sanger Sequencing

To analyze the mutation frequencies and spectrum of DNA modifications, target regions were amplified by PCR using gene-specific primers and genomic DNA extracted from leaves of non-transgenic and transgenic plants. Plant genomic DNA preparation was carried out using Qiagen DNeasy^®^ 96 Plant Kit (Qiagen, Hilden, Germany) according to the manufacturer’s instructions. PCR reaction was performed to amplify the genomic regions including sgRNA-targeting sequence using the gene-specific primers (Table S1). PCR conditions were as follows: 98 °C for 2 min and 35 cycles of 98 °C for 30 sec, 60 °C for 10 sec, and 72 °C for 30 sec. PCR products were purified using the QIAquick PCR purification kit (Qiagen, Germany) and used for further direct sequencing and cloning into the pGEM-T Easy vector (Promega, USA).

### 4.5. miRNA Detection Using Stem–Loop RT-PCR

Total RNA was extracted from leaves using TRIzol reagent (Invitrogen, Carlsbad, CA, USA) following a previously-described protocol [39]. For analyzing the level of mature miRNAs, stem–loop reverse transcription followed by RT-PCR was performed as described previously [40,41]. Next, 200 ng of total RNA was treated with RNAase-free DNase I (Promega, USA), and used for first-strand synthesis reactions using gene-specific RT primers and the Superscript III Reverse transcriptase (Invitrogen, USA). Reactions were performed at 16 °C for 45 min, followed by 60 cycles of 30 °C for 45 s, 42 °C for 45 s, and 50 °C for 1 s, in a 20 μL mixture containing 50 U of Superscript III RT (Invitrogen, USA), 4 U of RNaseOUT (Invitrogen, USA), and 1 μM stem–loop RT primer. The products were used for quantification of mature miRNA through qRT-PCR analysis with miRNA-specific forward and universal reverse primers. The rice U6 small nuclear RNA (snRNA) gene was used as an RNA loading control. A list of primers used in these experiments is available in Table S1.

### 4.6. Drought-Stress Treatments and Tolerance Evaluation

Thirty independent *OsmiR818b*-overexpressing transgenic lines were produced, and plants that grew normally without stunting were selected to eliminate the effects of somaclonal variations. Then, copy numbers of the transgenic plants were determined by TaqMan Q-PCR (Thermo Fisher, Waltham, MA, USA) using proves specific for the *bar* gene. Three independent single copy homozygous transgenic plants were further selected based on expression level and antibiotic segregation analysis. Nineteen independent transgenic plants expressing CRISPR/rCas9 with the sgRNA targeting *OsmiR818b* were produced to generate CRISPR/Cas9^miR818b^ mutants. The CRISPR/Cas9^miR818b^ #19 that contained monoallelic mutation (24bp deletion) on *OsmiR818b* locus was selected at the T_0_ stage. The plants were propagated, and the biallelic homozygous mutant of *OsmiR818b* was obtained at the T1 stage. T2 seeds of the homozygous mutant that contained Cas9 T-DNA were used for drought treatments. Non-transgenic plants (*O. Sativa* cv. Dongjin), *OsmiR818b*-overexpressing (T_3_ generation), and CRISPR/Cas9^miR818b^ (T_2_ stage)transgenic plants were sown on MS solid media and incubated in the dark growth chamber for two days at 28 °C. Two-day-old rice seedlings were transferred to the growth chamber with 16h light/ 8 h dark cycle for one additional day. Thirty seedlings from each line were transplanted into soil pots with a container and grown for an additional four weeks in a greenhouse (16h light/8h dark) at 30 °C. Drought stress was imposed by withholding water for 4 days. The plants were further incubated after re-watering for 6 days. Drought-induced symptoms were monitored by imaging transgenic and NT plants at the indicated time points using NEX-5N camera (Sony, Tôkyô, Japan).

## Figures and Tables

**Table 1:** List of target genes and mutation patterns in corresponding transgenic plants.

Target Gene	gRNA	GC(%)	T_m_	WT	Mono-Allelic	Biallelic		Total Plants	Mutation Rate (%)
						Homo	Hetero		
*miR399d*	TCACCAAAACGGCCTGCCAAAGG	55	73.0	40			6	46	13.0%
*miR418*	AATTCCACCGTGGTCCCTGGAGG	60	70.5	26		6	10	42	38.1%
*miR156d*	AGAGTGAGCACACGGCGTGATGG	60	70.1	3		8	37	48	93.8%
*miR399e*	TGCCCAGCAATGCAACTTTGCGG	50	69.6	14	14	1	3	32	56.3%
*miR399i*	TGCTAGCCTTTCCCTGCCAAAGG	55	69.2	3	6	8	4	21	85.7%
*miR169f*	AAGAGCTGATTCGGTAGCCAAGG	50	64.2	7	3	10	29	49	85.7%
*miR171f*	TTGGCATGGTTCAATCAAACCGG	40	63.7	21	1	3	24	49	57.1%
*OsNAC14*	AAGAGCTCTGGTGCAAGAAGGGG	50	61.8	3	4	5	36	48	93.8%
*miR156g*	GAAGAGAGTGAGCACACAGCGGG	55	61.0	1	2	3	12	18	94.4%
*miR399k*	GGTTACCAGACTACTGCCAAAGG	50	59.7		3	7	20	30	100.0%
*miR818b*	ATCCAAAATCCCTTATATTATGG	25	52.5	17	2			19	10.5%
*miR814a*	ACTTCATAGTACAACGAATCTGG	35	51.3	19	3		1	23	17.4%
*miR816*	ATATTTTACTACAACGAATCTGG	25	48.2	32	1			33	3.0%
**Total**				**186**	**39**	**51**	**182**	**458**	
				**40.6%**	**8.5%**	**11.1%**	**39.7%**		

## Supplementary Materials

Supplementary materials can be found at https://www.mdpi.com/1422-0067/21/24/9606/s1. Table S1: The primer list used in this study. Figure S1: Determination of mutation types from PCR products. Figure S2: Analysis of CRISPR/rCas9-mediated IN/Del patterns in CRISPR/rCas9*^OsNAC14^* transgenic plants.
