# Supplementary material for: Efficiency of Recombinant CRISPR/rCas9-Mediated miRNA Gene Editing in Rice
Source: Int J Mol Sci. 2020 Dec 16;21(24):9606. doi: 10.3390/ijms21249606 (PMC7766165; doi:10.3390/ijms21249606)
Supplement: Supplementary file 1 [file ijms-21-09606-s001.pdf]

Table S1. The primer list used in this study

Genes	Forward primer	Reverse primer	Experiments
OsU3 pro(HindIII) F	① AAGCTTAAGGAATCTTTAAACATACGA		For construction of CRISPR/rCas9
gRNA ter (XbaI) R	④ TCTAGAAAAACAAAAAAGCACCGACTCGGTGC		
miR399d	③ TCACCAAAACGGCCTGCCAAGTTTTAGAGCTAGAAATAGC	② TTGGCAGGCCGTTTTGGTGAGCCACGGATCATCTGCA	
miR418	③ AATTCCACCGTGGTCCCTGGGTTTTAGAGCTAGAAATAGC	② CCAGGGACCACGGTGGAATTGCCACGGATCATCTGCA	
miR156d	③ AGAGTGAGCACACGGCGTGAGTTTTAGAGCTAGAAATAGC	② TCACGCCGTGTGCTCACTCTGCCACGGATCATCTGCA	
miR399e	③ TGCCCAGCAATGCAACTTTGGTTTTAGAGCTAGAAATAGC	② CAAAGTTGCATTGCTGGGCAGCCACGGATCATCTGCA	
miR399i	③ TGCTAGCCTTTCCCTGCCAAGTTTTAGAGCTAGAAATAGC	② TTGGCAGGGAAAGGCTAGCAGCCACGGATCATCTGCA	
miR169f	③ AAGAGCTGATTCGGTAGCCAGTTTTAGAGCTAGAAATAGC	② TGGCTACCGAATCAGCTCTTGCCACGGATCATCTGCA	
miR171f	③ TTGGCATGGTTCAATCAAACGTTTTAGAGCTAGAAATAGC	② GTTTGATTGAACCATGCCAAGCCACGGATCATCTGCA	
OsNAC14	③ AAGAGCTCTGGTGCAAGAAGGTTTTAGAGCTAGAAATAGC	② GTTCTTGCACCAGAGCTCTTGCCACGGATCATCTGCA	
miR156g	③ GAAGAGAGTGAGCACACAGCGTTTTAGAGCTAGAAATAGC	② GCTGTGTGCTCACTCTCTTCGCCACGGATCATCTGCA	
miR399k	③ GGTTACCAGACTACTGCCAAGTTTTAGAGCTAGAAATAGC	② TTGGCAGTAGTCTGGTAACCGCCACGGATCATCTGCA	
miR818b	③ ATATTATGGGACGGAGGGATGTTTTAGAGCTAGAAATAGC	② ATCCCTCCGTCCCATAATATGCCACGGATCATCTGCA	
miR814a	③ ACTTCATAGTACAACGAATCGTTTTAGAGCTAGAAATAGC	② GATTCGTTGTACTATGAAGTGCCACGGATCATCTGCA	
miR816	③ ATATTTTACTACAACGAATCGTTTTAGAGCTAGAAATAGC	② GATTCGTTGTAGTAAAATATGCCACGGATCATCTGCA	
miR399d	(CACC) GCACAAGAGGCACACTAC	GTTGCCGCCCAGACTTCGTTTAC	Genomic DNA PCR<br>TA Cloning<br>PCR sequencing
miR418	(CACC) GGGTCACGGAAAAGGTC	GTGGGGATAACGATATTGGACCC	
miR156d	(CACC) GCTGAATTTCTCTGTACCAAG	CCGCTCACCGGATCCAAGAAG	
miR399e	(CACC) GGTTGGAAGAGGAGGAAG	GTCCAAAACACATATACAAGGACCG	
miR399i	(CACC) GCTGCTCAAGCATTGTCAG	GTACACCCTCAAGGCCTTAACTC	
miR169f	(CACC) CGTTGCAATCCATGGACATC	GGGGAGATATGGGTATCTAGGAC	
miR171f	(CACC) GTTTGCGTTCGCCATGTC	GGCAGGCATGTGAAATAACACGC	
OsNAC14	(CACC) CCTCCGACGAGCTTGTTCTGTG	CGGGTAACGCATGATTTGGGG	
miR156g	(CACC) GAGACCTCCCCAGATCTGG	TGAGGAGGAGCAGTAGTAGCCG	
miR399k	(CACC) AGAAAGGCCGTGTAGCTG	AGATTGCTCTCTCCCAATTCCTC	
miR818b	(CACC) GATCGATCTCGTCGTCG	GAACCTTGCACATGACTTCAGCTAG	
miR814a	(CACC) TTTCTGCCAGTGCTCCTAGC	CCCTCTGTGGTTTTAAGGGCAG	
miR816	(CACC) GATTCGTAGTACTAGAACGTG	GGCTGGCAGTGGCTCAGATC	
Universal_R	GTGCAGGGTCCGAGGT		Stem-loop RT-PCR
miR171f-5p_RT	GCGGCGGTGTTGGCATGGTTCAATC	GTCGTATCCAGTGCAGGGTCCGAGGTATTCGCACTGGATACGACTTTGATTG	
MiR818b_RT	GCGGCGGAATCCCTTATATTATGGG	GTCGTATCCAGTGCAGGGTCCGAGGTATTCGCACTGGATACGACCCGTCCCATA	

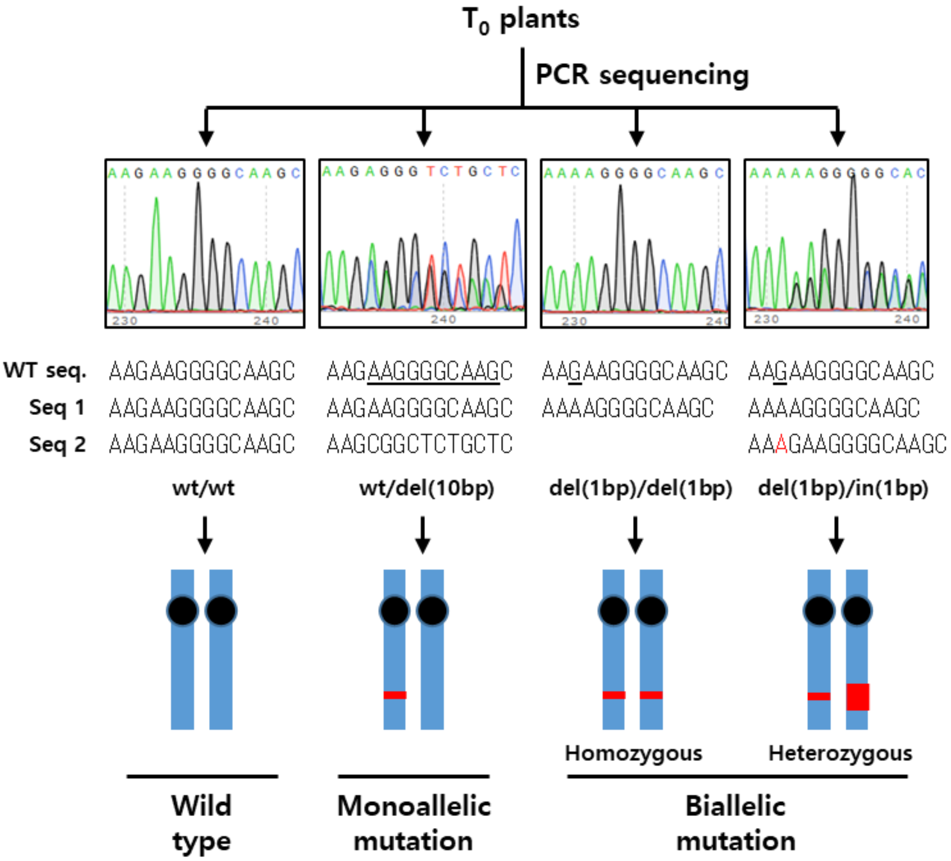

Figure S1. Determination of mutation types from PCR products. Genomic DNA extracted from T_0 transgenic plants was used for PCR-amplification of the single guide RNA (sgRNA) targeting site. The PCR products were analyzed by the Sanger sequencing method, and resultant chromatograms were used for the determination of mutation types. Wild type, non-mutated transgenic plants; Monoallelic mutation, one allele is mutated; Biallelic mutation, the two alleles are mutated; Homozygous, the two alleles have the same mutations; Heterozygous, the two alleles have different mutations.

(a)

CRISPR/rCAS9 *OsNAC14*

Sequence	In/Del	Freq.
AAGAGGAAGGCCGACGTGAAGAGCTCTGGTGCAAGAAGGGGCAAGCGGCTCTGCTCC	△0	13
AAGAGGAAGGCCGACGTGAAGAGCTCTGGTGC	△-71	1
AAGAGGAAGGC TCTGCTCC	△-38	1
AAGAGGAAGGCCGACGTCTGG	△-44/+4	1
AAGAGGAAGGCCGACGTGCTA	△-43/+3	4
AAGAGGAAGGCCCGGCTGCGGGA GGCAAGCGGCTCTGCTCC	△-27/+11	1
AAGAGGAAGGCCGACGTGAAGAGCTCTGGT CC	△-25	1
AAGAGGAAGGCCGACGTGAAGAGCTCTGGCAAA CGGCTCTGCTCC	△-16/+4	1
AAGAGGAAGGCCGACGTGAAGAGCTCTGGTGCAAG CGGCTCTGCTCC	△-10	1
AAGAGGAAGGCCGACGTGAAGAGCTCTGGT CAAGCGGCTCTGCTCC	△-11	1
AAGAGGAAGGCCGACGTGAAGAGCTCTGGTG GGCAAGCGGCTCTGCTCC	△-8	1
AAGAGGAAGGCCGACGTGAAGAGCTCTGGTAAG GGGGCAAGCGGCTCTGCTCC	△-8/+3	1
AAGAGGAAGGCCGACGTGAAGAGCTCTGGTG GGGCAAGCGGCTCTGCTCC	△-7	2
AAGAGGAAGGCCGACGTGAAGAGCTCTGGT GGGGCAAGCGGCTCTGCTCC	△-7	3
AAGAGGAAGGCCGACGTGAAGAGCTCTGGTGCAAAA CAAGCGGCTCTGCTCC	△-7/+1	1
AAGAGGAAGGCCGACGTGAAGAGCTCTGGTG GGGGCAAGCGGCTCTGCTCC	△-6	1
AAGAGGAAGGCCGACGTGAAGAGCTCTGGTGCAAA GCAAGCGGCTCTGCTCC	△-6/+1	1
AAGAGGAAGGCCGACGTGAAGAGCTCTGGTGCAAA GCAAGCGGCTCTGCTCC	△-6/+1	3
AAGAGGAAGGCCGACGTGAAGAGCTCTGGTGCAA GGCAAGCGGCTCTGCTCC	△-5	1
AAGAGGAAGGCCGACGTGAAGAGCTCTGGTGCAAG GCAAGCGGCTCTGCTCC	△-5	2
AAGAGGAAGGCCGACGTGAAGAGCTCTGGTGCAAAG GCAAGCGGCTCTGCTCC	△-5/+2	1
AAGAGGAAGGCCGACGTGAAGAGCTCTGGTGCAAAA GCAAGCGGCTCTGCTCC	△-5/+2	1
AAGAGGAAGGCCGACGTGAAGAGCTCTGGTGCG GGGGCAAGCGGCTCTGCTCC	△-5/+1	1
AAGAGGAAGGCCGACGTGAAGAGCTCTGGTGCA GGGGCAAGCGGCTCTGCTCC	△-4	2
AAGAGGAAGGCCGACGTGAAGAGCTCTGGTG AAAGGGGCAAGCGGCTCTGCTCC	△-4/+1	1
AAGAGGAAGGCCGACGTGAAGAGCTCTGGTGCAAA GGGCAAGCGGCTCTGCTCC	△-4/+1	5
AAGAGGAAGGCCGACGTGAAGAGCTCTGGTGCAAA GGGCAAGCGGCTCTGCTCC	△-4/+1	1
AAGAGGAAGGCCGACGTGAAGAGCTCTGGTGCAA GGGGCAAGCGGCTCTGCTCC	△-3	4
AAGAGGAAGGCCGACGTGAAGAGCTCTGGTGCAAA GGGGCAAGCGGCTCTGCTCC	△-3/+1	2
AAGAGGAAGGCCGACGTGAAGAGCTCTGGTGCA AAGGGGCAAGCGGCTCTGCTCC	△-2	2
AAGAGGAAGGCCGACGTGAAGAGCTCTGGTGCAA AGGGGCAAGCGGCTCTGCTCC	△-2	12
AAGAGGAAGGCCGACGTGAAGAGCTCTGGTGCAAG GGGGCAAGCGGCTCTGCTCC	△-2	4
AAGAGGAAGGCCGACGTGAAGAGCTCTGGTGCAAG GGGGGCAAGCGGCTCTGCTCC	△-2/+1	1
AAGAGGAAGGCCGACGTGAAGAGCTCTGGTGCAAGGGGGGGGCAAGCGGCTCTGCTCC	△-2/+3	1
AAGAGGAAGGCCGACGTGAAGAGCTCTGGTGCAAGG GGGGCAAGCGGCTCTGCTCC	△-2/+1	1
AAGAGGAAGGCCGACGTGAAGAGCTCTGGTGCAA AAGGGGCAAGCGGCTCTGCTCC	△-1	6
AAGAGGAAGGCCGACGTGAAGAGCTCTGGTGCAAGA GGGGCAAGCGGCTCTGCTCC	△-1	6
AAGAGGAAGGCCGACGTGAAGAGCTCTGGTGCA	△-1	1
AAGAGGAAGGCCGACGTGAAGAGCTCTGGTGCAAGTAAGGGGCAAGCGGCTCTGCTCC	△+1	1
AAGAGGAAGGCCGACGTGAAGAGCTCTGGTGCAAGGAAGGGGCAAGCGGCTCTGCTCC	△+1	1
AAGAGGAAGGCCGACGTGAAGAGCTCTGGTGCAAAGAAGGGGCAAGCGGCTCTGCTCC	△+1	1

Total 96 (48 plants)

(b)

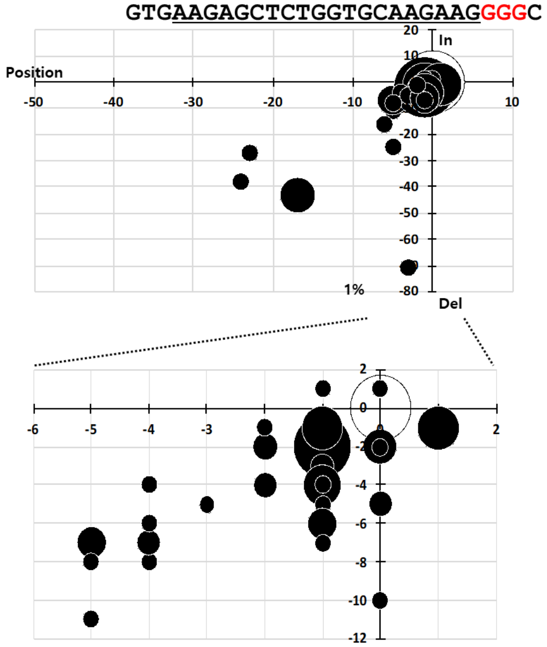

Figure S2. Analysis of CRISPR/rCas9-mediated base insertion and deletion (indel) patterns in CRISPR/rCas9*OsNAC14* transgenic plants. **a.** Indel mutation patterns and frequency. **b.** The graph showing the correlation between the degree of indel (y-axis) and the position where indel occurs (x-axis). The graph in the bottom is an enlarged image of the area where spots are concentrated. The white circle at the origin indicates the frequency of non-mutated plants.
